# Age-, sex-, and diagnosis-specific incidence rate of medically certified long-term sick leave among private sector employees: The Japan Epidemiology Collaboration on Occupational Health (J-ECOH) study

**DOI:** 10.1016/j.je.2017.01.003

**Published:** 2017-06-23

**Authors:** Chihiro Nishiura, Akiko Nanri, Ikuko Kashino, Ai Hori, Chihiro Kinugawa, Motoki Endo, Noritada Kato, Aki Tomizawa, Akihiko Uehara, Makoto Yamamoto, Tohru Nakagawa, Shuichiro Yamamoto, Toru Honda, Teppei Imai, Akiko Okino, Toshiaki Miyamoto, Naoko Sasaki, Kentaro Tomita, Satsue Nagahama, Takeshi Kochi, Masafumi Eguchi, Hiroko Okazaki, Taizo Murakami, Chii Shimizu, Makiko Shimizu, Isamu Kabe, Tetsuya Mizoue, Tomofumi Sone, Seitaro Dohi

**Affiliations:** a Department of Safety and Health, Tokyo Gas Co., Ltd., Tokyo, Japan; b Department of Epidemiology and Prevention, Center for Clinical Sciences, National Center for Global Health and Medicine, Tokyo, Japan; c Department of Public Health, Tokyo Women's Medical University, Tokyo, Japan; d Fuji Electric Co., Ltd., Tokyo, Japan; e Fuji Electric Co., Ltd., Kanagawa, Japan; f Yamaha Corporation, Shizuoka, Japan; g Hitachi, Ltd., Ibaraki, Japan; h Azbil Corporation, Tokyo, Japan; i Nippon Steel & Sumitomo Metal Corporation Kimitsu Works, Chiba, Japan; j Mitsubishi Fuso Truck and Bus Corporation, Kanagawa, Japan; k Mitsubishi Plastics, Inc., Tokyo, Japan; l All Japan Labour Welfare Foundation, Tokyo, Japan; m Furukawa Electric Co., Ltd., Tokyo, Japan; n Mitsui Chemicals, Inc., Tokyo, Japan; o Mizue Medical Clinic, Keihin Occupational Health Center, Kanagawa, Japan; p National Institute of Public Health, Saitama, Japan

**Keywords:** Incidence, Mental disorders, Neoplasms, Occupational health, Sick leave

## Abstract

**Background:**

Long-term sick-leave is a major public health problem, but data on its incidence in Japan are scarce. We aimed to present reference data for long-term sick-leave among private sector employees in Japan.

**Methods:**

The study population comprised employees of 12 companies that participated in the Japan Epidemiology Collaboration on Occupational Health Study. Details on medically certified sick-leave lasting ≥30 days were collected from each company. Age- and sex-specific incidence rate of sick-leave was calculated for the period of April 2012 to March 2014.

**Results:**

A total of 1422 spells in men and 289 in women occurred during 162,989 and 30,645 person-years of observation, respectively. The three leading causes of sick-leave (percentage of total spells) were mental disorders (52%), neoplasms (12%), and injury (8%) for men; and mental disorders (35%), neoplasms (20%), and pregnancy-related disease (14%) for women. Incidence rate of sick-leave due to mental disorders was relatively high among men in their 20s–40s but tended to decrease with age among women. Incidence rate of sick-leave due to neoplasms started to increase after age 50 in men and after age 40 in women, making neoplasms the leading cause of sick-leave after age 50 for women and after age 60 for men and the second leading cause after age 40 for women and after age 50 for men. Pregnancy-related disease was the second leading cause of sick-leave among women aged 20–39 years.

**Conclusions:**

These results suggest that mental disorder, neoplasms, and pregnancy-related disease are the major causes of long-term sick-leave among private sector employees in Japan.

## Highlights

• Incidence rate of sick-leave lasting ≥30 days was studied in 12 Japanese companies.
• The major causes were mental disorder, neoplasms, and pregnancy-related disease.
• Mental disorder and pregnancy-related disease were common in women aged 30–39 years.

## Introduction

Long-term sick-leave is regarded as a public health problem, and increasing attention is being focused on its relationship with future health status^1^ and mortality.^2^ While data on incidence of long-term sick-leave can facilitate the prevention and management of sick-leave, several methodological issues in research regarding long-term sick-leave need to be addressed. For example, self-reported sick-leave is imprecise in cases of leave exceeding 1 week, and not self-reported but medically certified diagnosis leading to sick-leave requests are regarded as reliable in the recent studies on sick-leave; thus, objective data, such as company-based data, are preferable for accurately evaluating long-term sick-leave.^3^, ^4^ Additionally, incidence research requires a clearly defined population from which sick-leave cases arise.^5^

Qualified incidence studies have been performed in a few countries. For instance, in an international comparison study of incidence of medically-certified sick-leave due to mental disorders,^4^ which is the leading cause of sick-leave in most high-income countries,^6^ a total of 10 studies were identified in Finland, Norway, the Netherlands, Canada, and Brazil. However, no such studies have been conducted in Asia. Studying incidence of sick-leave is particularly challenging in countries without a sick-leave registry, such as Japan. Since 2012, the Japan Epidemiology Collaboration on Occupational Health (J-ECOH) Study group has been collecting company-based sick-leave records from a number of private companies in Japan. The J-ECOH Study population comprises both male and female workers across the entire working age span.

In this study, we aimed to present reference data for long-term sick-leave among private sector employees in Japan using data from this large-scale multicenter occupational cohort.

## Methods

### Study population

The Japan Epidemiology Collaboration on Occupational Health Study (J-ECOH) is an ongoing multi-center occupational cohort study in Japan.^7^, ^8^ As of March 2015, a total of 12 private sector companies, mainly in the manufacturing industry, provided official records of medically certified sick-leave to the J-ECOH Study and reported the number of employees by sex- and 5-year age group from April 1, 2012 through March 31, 2014, which formed the data of the current study. The total numbers of male and female employees aged 20–64 years were 82,510 and 15,475 in 2012 and 81,316 and 15,313 in 2013, respectively.

### Survey of sick-leave

In Japan, where paid sick-leave is not stipulated by law, paid sick-leave schemes vary among companies. At the companies participating in the J-ECOH Study, employees were entitled to paid sick-leave with over two-thirds of the salary for at least 18 months, and job security was guaranteed for at least 30 months. Sick-leave data obtained in the J-ECOH Study included date of birth, sex, start and end dates of sick-leave, and the subject's diagnosis. All diagnoses for sick-leave were based on the medical certificate written by the attending physician (i.e., a general practitioner or specialist, but not an occupational physician), which had been submitted to the company by the employee when applying for paid sick-leave. In the present study, we analyzed instances of medically certified sick-leave that started between April 1, 2012 and March 31, 2014 and lasted 30 days or more. Subjects who filed for sick-leave that started during March 2014 were followed until April 30, 2014 to determine whether the absence lasted 30 days or more. In this study, long-term sick-leave was defined as sick-leave lasting 30 days or more.

### International Classification of Diseases 10th revision coding

We classified diagnoses according to the International Classification of Diseases, 10th revision (ICD-10), with reference to the Japanese standard disease-code master.^9^ Of the 1711 certificates, the 1273 that completely matched an ICD-10 classification were mechanically encoded by text matching using Microsoft Excel (Microsoft Corporation, Redmond, WA, USA). The remaining 438 unmatched certificates were manually and independently encoded by two occupational physicians of the J-ECOH Study group (CN and CK) with reference to the master; of these, both physicians agreed upon the coding for 370 certificates. The disagreements for the remaining 68 certificates between the two physicians were mainly due to multiple diagnoses. As we were unable to obtain original clinical record to determine the primary diagnosis, another occupational physician of the J-ECOH Study group (AH) independently coded and ultimately decided on their ICD-10 code.

### Definition of incidence rate

There are wide variations in the definition of sick-leave incidence, which hamper the comparison among studies. Hensing proposed an index of sick-leave incidence, which would provide comparable data.^10^ In this study, we used this method in calculating sick-leave incidence as follows:

$$\text{Incidence}\;\text{rate}=\frac{\text{Number}\;\text{of}\;\text{new}\;\text{sick}\text{-}\text{leave}\;\text{spells}\;\text{during}\;\text{study}\;\text{period}}{\text{Time}\;\text{each}\;\text{person}\;\text{was}\;\text{at}\;\text{risk}\;\text{for}\;\text{new}\;\text{sick}\text{-}\text{leave,}\;\text{totaled}\;\text{for}\;\text{all}\;\text{persons }}$$

Multiple sick-leave spells for the same person were counted separately. Incidence rate was presented per 1000 person-years. We defined a person who was at risk of sick leave as present employees. Absence duration (time in current and new spells of sick-leave) during the observation period was subtracted from the time at risk.

Person-years of observation were 162,989 for men and 30,645 for women. Data management and aggregation were performed using Stata14 (Stata Corporation, College Station, TX, USA), and incidence rate calculations were performed using Excel 2010.

### Ethical approval

This study was approved by the Institutional Review Board of the National Center for Global Health and Medicine (NCGM-G-001140-07). All data were analyzed anonymously.

## Results

Table 1, Table 2 show the number of spells and incidence rate of sick-leave by ICD-10 chapters for men and women, respectively. A total of 1711 spells (1422 for men and 289 for women) occurred during the 2-year observation period. The top five primary categories of sick-leave spells (percentage of total spells) were mental disorders (ICD-10:F00-F99; 52%), neoplasms (ICD-10:C00-D48; 12%), injury (ICD-10:S00-T98; 8%), circulatory disease (ICD-10:I00-I99; 7%), and musculoskeletal disease (ICD-10:M00-M99; 7%) for men; corresponding categories for women were mental disorders (35%), neoplasms (20%), pregnancy-related disease (ICD-10:O00-O99; 14%), injury (9%), and musculoskeletal disease (7%). Age-specific major causes of sick-leave (percentage of total spells in each sex- and age-group) were mental disorders among men in their 20s–40s (20s, 73%; 30s, 71%; 40s, 62%), mental disorders (34%) and neoplasms (22%) among men in their 50s, neoplasms (35%) among men aged 60–64 years, mental disorders (20s, 58%; 30s, 35%) and pregnancy-related disease (20s, 19%; 30s, 32%) among women in their 20s–30s, mental disorders (38%) and neoplasms (27%) among women in their 40s, and neoplasms (50s, 33%; 60s, 58%) among women aged 50–64.

**Table 1:** Incidence rate of sick-leave among men in the J-ECOH cohort, 2012–2013.

ICD-10 chapters	Number of spells during 2 years						Incidence rate of sick-leave (new spells/1000 person-years)					
	Overall	Age categories, years					Overall	Age categories, years				
		20–29	30–39	40–49	50–59	60–64		20–29	30–39	40–49	50–59	60–64
Overall	1422	187	231	457	433	114	8.7	8.0	6.6	9.0	10.7	8.6
Certain infectious and parasitic diseases (A00-B99)	12	0	1	5	4	2	0.1	0.0	0.0	0.1	0.1	0.2
Neoplasms (C00-D48)	176	0	15	28	93	40	1.1	0.0	0.4	0.6	2.3	3.0
Diseases of the blood and blood-forming organs and certain disorders (D50-89)	1	0	0	0	1	0	0.0	0.0	0.0	0.0	0.0	0.0
Endocrine, nutritional and metabolic diseases (E00-E90)	20	0	1	7	7	5	0.1	0.0	0.0	0.1	0.2	0.4
Mental and behavioural disorders (F00-F99)	736	136	165	283	146	6	4.5	5.8	4.7	5.6	3.6	0.5
Diseases of the nervous system (G00-G99)	53	8	7	21	13	4	0.3	0.3	0.2	0.4	0.3	0.3
Diseases of the eye and adnexa (H00-H59)	16	2	3	3	5	3	0.1	0.1	0.1	0.1	0.1	0.2
Diseases of the ear and mastoid process (H60-95)	4	0	0	3	0	1	0.0	0.0	0.0	0.1	0.0	0.1
Diseases of the circulatory system (I00-I99)	104	2	2	27	58	15	0.6	0.1	0.1	0.5	1.4	1.1
Diseases of the respiratory system (J00-J99)	16	2	3	4	6	1	0.1	0.1	0.1	0.1	0.1	0.1
Diseases of the digestive system (K00-K93)	38	2	6	7	17	6	0.2	0.1	0.2	0.1	0.4	0.5
Diseases of the skin and subcutaneous tissue (L00-L99)	5	1	2	1	0	1	0.0	0.0	0.1	0.0	0.0	0.1
Diseases of the musculoskeletal system and connective tissue (M00-M99)	102	10	10	29	41	12	0.6	0.4	0.3	0.6	1.0	0.9
Diseases of the genitourinary system (N00-N99)	7	1	1	2	2	1	0.0	0.0	0.0	0.0	0.0	0.1
Congenital malformations, deformations and chromosomal abnormalities (Q00-Q99)	2	0	0	1	1	0	0.0	0.0	0.0	0.0	0.0	0.0
Symptoms, signs and abnormal clinical and laboratory findings, not elsewhere classified (R00-R99)	7	1	0	4	2	0	0.0	0.0	0.0	0.1	0.0	0.0
Injury, poisoning and certain other consequences of external causes (S00-T98)	118	22	14	30	36	16	0.7	0.9	0.4	0.6	0.9	1.2
Factors influencing health status and contact with health services	4	0	1	1	1	1	0.0	0.0	0.0	0.0	0.0	0.1
N/A	1	0	0	1	0	0	0.0	0.0	0.0	0.0	0.0	0.0
Risk time (person-years)	162,989	23,488	35,070	50,813	40,379	13,240						

N/A, no applicable ICD-10 code for the medically certified diagnosis.

Categories were omitted when there was no case.

**Table 2:** Incidence rate of sick-leave among women in the J-ECOH cohort, 2012–2013.

ICD-10 chapters	Number of spells during 2 years						Incidence rate of sick-leave (new spells/1000 person-years)					
	Overall	Age categories, years					Overall	Age categories, years				
		20–29	30–39	40–49	50–59	60–64		20–29	30–39	40–49	50–59	60–64
Overall	289	52	84	93	48	12	9.4	11.3	11.1	8.9	7.7	6.5
Certain infectious and parasitic diseases (A00-B99)	1	0	0	0	1	0	0.0	0.0	0.0	0.0	0.2	0.0
Neoplasms (C00-D48)	58	3	7	25	16	7	1.9	0.7	0.9	2.4	2.6	3.8
Diseases of the blood and blood-forming organs and certain disorders involving the immune mechanism (D50-89)	1	0	0	1	0	0	0.0	0.0	0.0	0.1	0.0	0.0
Endocrine, nutritional and metabolic diseases (E00-E90)	2	0	1	0	1	0	0.1	0.0	0.1	0.0	0.2	0.0
Mental and behavioural disorders (F00-F99)	101	30	29	35	7	0	3.3	6.5	3.8	3.4	1.1	0.0
Diseases of the nervous system (G00-G99)	6	2	2	1	1	0	0.2	0.4	0.3	0.1	0.2	0.0
Diseases of the eye and adnexa (H00-H59)	3	0	0	0	2	1	0.1	0.0	0.0	0.0	0.3	0.5
Diseases of the ear and mastoid process (H60-95)	2	1	1	0	0	0	0.1	0.2	0.1	0.0	0.0	0.0
Diseases of the circulatory system (I00-I99)	8	0	1	4	2	1	0.3	0.0	0.1	0.4	0.3	0.5
Diseases of the respiratory system (J00-J99)	3	0	1	0	2	0	0.1	0.0	0.1	0.0	0.3	0.0
Diseases of the digestive system (K00-K93)	9	0	3	3	2	1	0.3	0.0	0.4	0.3	0.3	0.5
Diseases of the skin and subcutaneous tissue (L00-L99)	2	1	0	0	1	0	0.1	0.2	0.0	0.0	0.2	0.0
Diseases of the musculoskeletal system and connective tissue (M00-M99)	21	1	4	9	6	1	0.7	0.2	0.5	0.9	1.0	0.5
Diseases of the genitourinary system (N00-N99)	2	1	0	1	0	0	0.1	0.2	0.0	0.1	0.0	0.0
Pregnancy, childbirth and the puerperium (O00-O99)	41	10	27	4	0	0	1.3	2.2	3.6	0.4	0.0	0.0
Congenital malformations, deformations and chromosomal abnormalities (Q00-Q99)	1	0	0	0	1	0	0.0	0.0	0.0	0.0	0.2	0.0
Symptoms, signs and abnormal clinical and laboratory findings, not elsewhere classified (R00-R99)	1	0	0	1	0	0	0.0	0.0	0.0	0.1	0.0	0.0
Injury, poisoning and certain other consequences of external causes (S00-T98)	27	3	8	9	6	1	0.9	0.7	1.1	0.9	1.0	0.5
Risk time (person-years)	30,645	4615	7570	10,419	6201	1840						

Categories were omitted when there was no case.

Overall incidence rate of all-cause sick-leave (new spells/1000 person-years) was 8.7 in men and 9.4 in women. The incidence rate of all-cause sick-leave in men was lowest among those in their 30s (6.6 spells/1000 person-years) and highest among those in their 50s (10.7 spells/1000 person-years), while that in women was highest among those in their 20s (11.3 spells/1000 person-years) and thereafter tended to decrease with age (6.5 spells/1000 person-years among those in their 60s). Incidence rate of sick-leave due to mental disorders was relatively high among men in their 20s–40s and declined among men aged 50 or older; in women, this rate tended to decrease with age. Sick-leave due to neoplasms began to rise from age 50 among men and from age 40 among women. In women, incidence rate of sick-leave due to pregnancy-related disease peaked at 3.6 spells/1000 person-years in their 30s, a rate similar to that for mental disorders in the same age group (3.8 spells/1000 person-years).

Table 3, Table 4 present incidence rate of sick-leave due to mental disorders and neoplasms for men and women, respectively. Main diagnoses for mental disorders (percentage of total spells due to mental disorders) were mood disorders (ICD-10:F30-F39; men 69%; women 53%) and neurotic, stress-related, and somatoform disorders (ICD-10:F40-F48; men 25%; women 39%). Incidence rate of sick-leave for mood disorders was relatively high among men in their 20s–40s but decreased among those in their 50s or older; in contrast, incidence tended to decrease steadily with age among women. Incidence rate of sick-leave for neurotic, stress-related, and somatoform disorders decreased with age in both men and women. With regard to neoplasms, incidence rate of sick-leave among men started to increase from age 50, mainly due to malignant neoplasms of digestive organs (ICD-10:C15-C26; including stomach and colorectum), and respiratory and intrathoracic organs (ICD-10:C30-C39; including lung), whereas among women, incidence started to increase in their 40s, mainly due to malignant neoplasms of breast (ICD-10:C50-C50) and female genital organs (ICD-10:C51-C58), in addition to digestive organs. Among women, 28% of neoplasms (16 of 58) were of uncertain or unknown behavior (ICD-10:D37-D48).

**Table 3:** Incidence rate of sick-leave due to neoplasms or mental and behavioural disorders among men in the J-ECOH cohort, 2012–2013.

ICD-10 chapters and blocks	Number of spells during 2 years						Incidence rate of sick-leave (new spells/1000 person-years)					
	Overall	Age categories, years					Overall	Age categories, years				
		20–29	30–39	40–49	50–59	60–64		20–29	30–39	40–49	50–59	60–64
Neoplasms (C00-D48)	176	0	15	28	93	40	1.1	0.0	0.4	0.6	2.3	3.0
Malignant neoplasms (C00-C97)	156	0	9	23	88	36	1.0	0.0	0.3	0.5	2.2	2.7
Malignant neoplasms of lip, oral cavity and pharynx (C00-C14)	8	0	0	2	3	3	0.0	0.0	0.0	0.0	0.1	0.2
Malignant neoplasms of digestive organs (C15-C26)	78	0	3	10	50	15	0.5	0.0	0.1	0.2	1.2	1.1
Malignant neoplasms of respiratory and intrathoracic organs (C30-C39)	32	0	3	4	17	8	0.2	0.0	0.1	0.1	0.4	0.6
Melanoma and other malignant neoplasms of skin (C43-C44)	1	0	0	0	1	0	0.0	0.0	0.0	0.0	0.0	0.0
Malignant neoplasms of male genital organs (C60-C63)	6	0	0	1	2	3	0.0	0.0	0.0	0.0	0.0	0.2
Malignant neoplasms of urinary tract (C64-C68)	6	0	0	1	2	3	0.0	0.0	0.0	0.0	0.0	0.2
Malignant neoplasms of eye, brain and other parts of central nervous system (C69-C72)	1	0	0	0	0	1	0.0	0.0	0.0	0.0	0.0	0.1
Malignant neoplasms of ill-defined, secondary and unspecified sites (C76-C80)	3	0	0	0	3	0	0.0	0.0	0.0	0.0	0.1	0.0
Malignant neoplasms, stated or presumed to be primary, of lymphoid, haematopoietic and related tissue (C81-C96)	21	0	3	5	10	3	0.1	0.0	0.1	0.1	0.2	0.2
Benign neoplasms (D10-D36)	1	0	1	0	0	0	0.0	0.0	0.0	0.0	0.0	0.0
Neoplasms of uncertain or unknown behaviour (D37-D48)	19	0	5	5	5	4	0.1	0.0	0.1	0.1	0.1	0.3
Mental and behavioural disorders (F00-F99)	736	136	165	283	146	6	4.5	5.8	4.7	5.6	3.6	0.5
Organic, including symptomatic, mental disorders (F00-F09)	1	0	0	0	1	0	0.0	0.0	0.0	0.0	0.0	0.0
Mental and behavioural disorders due to psychoactive substance use (F10-F19)	8	2	1	4	1	0	0.0	0.1	0.0	0.1	0.0	0.0
Schizophrenia, schizotypal and delusional disorders (F20-F29)	22	3	6	9	4	0	0.1	0.1	0.2	0.2	0.1	0.0
Mood [affective] disorders (F30-F39)	510	80	112	200	115	3	3.1	3.4	3.2	3.9	2.8	0.2
Neurotic, stress-related and somatoform disorders (F40-F48)	183	49	45	62	24	3	1.1	2.1	1.3	1.2	0.6	0.2
Behavioural syndromes associated with physiological disturbances and physical factors (F50-F59)	1	1	0	0	0	0	0.0	0.0	0.0	0.0	0.0	0.0
Disorders of adult personality and behaviour (F60-F69)	1	0	0	1	0	0	0.0	0.0	0.0	0.0	0.0	0.0
Disorders of psychological development (F80-F89)	2	1	0	1	0	0	0.0	0.0	0.0	0.0	0.0	0.0
Unspecified mental disorder (F99-F99)	8	0	1	6	1	0	0.0	0.0	0.0	0.1	0.0	0.0

Categories were omitted when there was no case.

**Table 4:** Incidence rate of sick-leave due to neoplasms or mental and behavioural disorders among women in the J-ECOH cohort, 2012–2013.

ICD-10 chapters and blocks	Number of spells during 2 years						Incidence rate of sick-leave (new spells/1000 person-years)					
	Overall	Age categories, years					Overall	Age categories, years				
		20–29	30–39	40–49	50–59	60–64		20–29	30–39	40–49	50–59	60–64
Neoplasms (C00-D48)	58	3	7	25	16	7	1.9	0.7	0.9	2.4	2.6	3.8
Malignant neoplasms (C00-C97)	34	2	2	11	15	4	1.1	0.4	0.3	1.1	2.4	2.2
Malignant neoplasms of lip, oral cavity and pharynx (C00-C14)	1	0	0	0	1	0	0.0	0.0	0.0	0.0	0.2	0.0
Malignant neoplasms of digestive organs (C15-C26)	8	0	1	2	3	2	0.3	0.0	0.1	0.2	0.5	1.1
Malignant neoplasms of respiratory and intrathoracic organs (C30-C39)	2	0	0	0	2	0	0.1	0.0	0.0	0.0	0.3	0.0
Malignant neoplasm of breast (C50-C50)	10	0	0	4	5	1	0.3	0.0	0.0	0.4	0.8	0.5
Malignant neoplasms of female genital organs (C51-C58)	9	0	1	4	4	0	0.3	0.0	0.1	0.4	0.6	0.0
Malignant neoplasms, stated or presumed to be primary, of lymphoid, haematopoietic and related tissue (C81-C96)	4	2	0	1	0	1	0.1	0.4	0.0	0.1	0.0	0.5
Benign neoplasms (D10-D36)	8	0	3	5	0	0	0.3	0.0	0.4	0.5	0.0	0.0
Neoplasms of uncertain or unknown behaviour (D37-D48)	16	1	2	9	1	3	0.5	0.2	0.3	0.9	0.2	1.6
Mental and behavioural disorders (F00-F99)	101	30	29	35	7	0	3.3	6.5	3.8	3.4	1.1	0.0
Schizophrenia, schizotypal and delusional disorders (F20-F29)	5	1	3	1	0	0	0.2	0.2	0.4	0.1	0.0	0.0
Mood [affective] disorders (F30-F39)	54	18	18	12	6	0	1.8	3.9	2.4	1.2	1.0	0.0
Neurotic, stress-related and somatoform disorders (F40-F48)	39	11	8	20	0	0	1.3	2.4	1.1	1.9	0.0	0.0
Behavioural syndromes associated with physiological disturbances and physical factors (F50-F59)	2	0	0	1	1	0	0.1	0.0	0.0	0.1	0.2	0.0
Unspecified mental disorder (F99-F99)	1	0	0	1	0	0	0.0	0.0	0.0	0.1	0.0	0.0

Categories were omitted when there was no case.

## Discussion

As a whole, the first- and second-leading causes of long-term sick-leave were mental disorders and neoplasms, respectively, which together accounted for more than half of sick-leave episodes lasting 30 days or more. By age and sex, major causes of incidence of long-term sick-leave were mental disorders in men aged 20–59 and women aged 20–49, neoplasms in women aged 40–64 and men aged 50–64, and pregnancy-related disease in women aged 20–39. This study is the first large-scale study of age-, sex-, and diagnosis-specific incidence rates of medically certified long-term sick-leave among private sector employees in Japan.

In this study, mental disorder was the leading cause of sick-leave among both men and women, with mood disorder the most frequent subtype. Additionally, men in their 30s or later were more likely to take long-term sick-leave due to mood disorder than women in the same age group. This finding appears to conflict with the fact that in a Dutch study among company employees aged 20–59, medically certified sick-leave lasting 28 days or more due to common mental disorders, including depressive and anxiety disorders, occurred more often in women than in men across all age groups.^11^ In general, women are more likely to experience a mood disorder than men.^12^ Although we lack any plausible reason for this lower rate of sick-leave due to mood disorders in Japanese women than that in Japanese men, we speculate that women are more likely than men to quit a job, rather than take long-term sick leave, once they are diagnosed with a mood disorder.

Incidence rate of sick-leave due to neoplasms started to increase from age 50 among men and from age 40 among women, with women's incidence rates being consistently higher than those of men (Table 3, Table 4). The age distribution of cancer-related sick-leave was compatible with onset age of cancer in the Japanese general population, according to National Cancer Registry data from 2011.^13^ In the present long-term sick-leave registry, major cancer sites were digestive and respiratory organs in men and breast, genital, and digestive organs in women. According to the 2011 National Cancer Registry data, major cancers in the general working-age population (20–64 years) were stomach, colorectal, lung, and prostate cancers in men and breast, uterus, colorectal, and stomach cancers in women.^13^ The only major cancer site not accounted for in our data was the prostate, cancer of which tends not to require long-term sick-leave.^14^

Notably in women, 19% and 32% of long-term sick-leave taken by women in their 20s and 30s, respectively, was due to pregnancy-related disease, such as threatened premature labor, hyperemesis gravidarum, and threatened abortion. This finding is consistent with data showing that risk of complications in pregnancy increases with advancing age^15^ and that mean maternal age at first childbirth has risen over time, exceeding age 30 since 2011 in Japan.^16^ Our findings regarding sick-leave point to a need for support and protection of pregnant women in the workplace.

The present study has several strengths that warrant mention. The sample size is relatively large for a sick-leave study in Japan, with a source population of approximately 100,000 employees. In addition, this study used sick-leave data from official company records, which is free from recall bias^17^, ^18^, ^19^ and based on a highly reliable physician's diagnosis. However, limitations to our study also deserve mention. First, the present study was conducted in large-scale companies, so caution is required when generalizing the study findings to small- or middle-scale companies. Specifically, large-scale companies often have generous sickness insurance schemes, such as long-term sick-leave compensation, which may contribute to the increase of sick-leave incidence.^5^ Therefore, sick-leave incidence in small- or middle-scale companies without such a system would be lower than the present estimate because of higher retirement rate among those who develop a serious disease. Second, worries about stigma due to the actual diagnosis may prompt a physician to describe a different diagnosis on the application form for paid sick-leave. Third, sick-leave incidence in their 60s was lower than that in their 50s. This could be ascribed, at least in part, to the selection of workers according to health status at re-employment; unhealthy workers were less likely to be re-employed after retirement than healthy workers. Finally, interpretation of the results for women requires careful attention, given their relatively small sample size in the present study, particularly those aged in their 20s and 60s.

In conclusion, major causes of incidence of long-term sick-leave in Japanese private sector employees were mental disorders, followed by neoplasms. Pregnancy-related disease was also common in women aged 20–39 years. These descriptive data will help occupational health professionals, human resource managers, and policy makers in the development of strategies to prevent and manage long-term sick-leave.

## Conflicts of interest

None declared.
